# An Integrated Energy-Efficient Wireless Sensor Node for the Microtremor Survey Method

**DOI:** 10.3390/s19030544

**Published:** 2019-01-28

**Authors:** Ruyun Tian, Longxu Wang, Xiaohua Zhou, Hao Xu, Jun Lin, Linhang Zhang

**Affiliations:** 1 College of Instrumentation and Electrical Engineering, Jilin University, Changchun 130061, China; tianry18@mails.jlu.edu.cn (R.T.); wanglx17@mails.jlu.edu.cn (L.W.); haoxu17@mails.jlu.edu.cn (H.X.); 2 Key Laboratory of Geophysical Exploration Equipment, Ministry of Education, Jilin University, Changchun 130061, China; linxing@jlu.edu.cn

**Keywords:** microtremor survey method, energy-efficient, 4G wireless monitoring system, ambient-noise measurement, strata velocity structure

## Abstract

The microtremor survey method (MSM) has the potential to be an important geophysical method for identifying the strata velocity structure and detecting the buried fault structures. However, the existing microtremor exploration equipment has been unable to satisfy the requirements of the MSM, which suffers from low data accuracy, long measurement time, and blind acquisition. In this study, we combined a 2 Hz moving coil geophone with advanced acquisition systems to develop a new integrated energy-efficient wireless sensor node for microtremor exploration. A high-precision AD chip and noise matching technology are used to develop a low-noise design for the sensor node. Dynamic frequency selection technology (DFS) and dynamic power management technology (DPM) are used to design an energy-efficient mode. The data quality monitoring system solves the closed technical flaws between the acquisition systems and the control center via 4G wireless monitoring technologies. According to the results of a series of in situ tests and field measurements, the noise level of the system was 0.7 μV@500 Hz with 0 dB attenuation and 220 mW power consumption of the system in the autonomous data acquisition mode. Therefore, it provides substantial support for the effective data acquisition over long measurement durations in microtremor exploration processes. The applicability of the system is evaluated using field data, according to which the integrated energy-efficient wireless sensor node is convenient and effective for MSM.

## 1. Introduction

The traditional seismic exploration method makes use of the reflection or refraction at the wave impedance interface during the seismic wave transmission process of underground media. In urban or other areas that have complex geological conditions, the use of conventional geological drilling and traditional geophysical exploration methods is severely limited due to high construction costs. Various boreholes cannot be implemented and there will be blind spots of geological information due to the presence of underground pipelines and existing buildings. Compared with traditional geophysical prospecting methods, microtremor survey method (MSM) uses the ambient noise of the earth or man-made noise without an artificial source. Thus, it is a new noninvasive exploration method with a wide range of potential applications.

**Figure 1:**
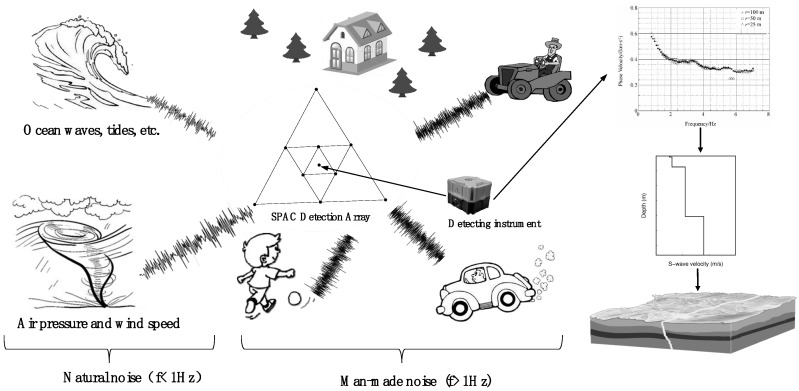
Schematic diagram of microtremor survey method (MSM).

Microtremor signals are composed mainly of body waves (primary and secondary waves) and surface waves (Rayleigh and Love waves) and the energy of the surface wave is the main component (more than 70 %) of the total energy [1]. This type of vibration phenomenon is extremely irregular in time and space. It is caused by two types of sources: Natural noise, such as noise due to air pressure, wind speed, ocean wave, and tidal changes (signal frequency <1 Hz), and noise from vehicle driving, machine operation, and people’s daily production activities (signal frequency >1 Hz). A schematic diagram of MSM is shown in Figure 1. Although the amplitude and shape of the microtremor signal changed over time and space, there is statistical stability within a range, which can be described by a stationary stochastic process in time and space [2]. The spatial autocorrelation method (SPAC) in the microtremor survey was proposed by Aki in 1957 and according to its array arrangement method, the original geophones are arranged in circles or nested triangles, as shown in Figure 1. In addition, the extended spatial autocorrelation method (ESPAC) has been developed, in which the geophones are freely arranged. These methods, which are founded on the same principle, are collectively called the (SPAC). MSM is based on the theory of stationary stochastic processes. The natural field source microtremor signals are observed by the SPAC array, data processing, and analysis technology are used to extract the surface wave (Rayleigh wave) dispersion information, and an inversion technique is used to obtain the S-wave velocity structure for the underground medium [3].

Determination of the S-wave velocity structure for imaging and monitoring the structure of the subsurface via MSM has become a new research hotspot in geophysical exploration. Aki first used the spatial autocorrelation method to extract the surface wave dispersion curve from the microtremor signal in 1957 [4]. Then, the extended spatial autocorrelation method for the complex array improved the efficiency of microtremor data processing [5,6]. MSM has realized satisfactory application results in the mapping of deeply buried fault structures for geothermal reservoir exploration [7,8,9]. Additional successful fracture zone detection examples have been demonstrated in underground exploration in complex mountainous areas or cities [10]. 

The equipment that is currently used by MSM is divided into three main categories: The first device type focuses on the vibration signals of lower frequencies for medium-deep exploration, such as the cable-free self-positioning seismographs of Jilin University of China and the MTKV-1C seismometer of Japan [11,12]. The data collection methods of these instruments are blind, e.g., they are unable to timely identify invalid records, which affects the operational efficiency, and they have closed technical defects. However, many application domains require that sensor nodes be deployed in harsh environments, such as on the jungle or in mountain areas, making these nodes more prone to failures. Sensor node failures can be catastrophic for microtremor survey [13,14], such as the inability to find invalid records in time, affecting operational efficiency, and having closed technical defects. To adapt to such conditions, sensor networks often adopt fault monitoring techniques to eliminate the need for unnecessary redesign [15]. In addition, these instruments cannot accommodate long-term real-time monitoring due to insufficient power supply. The second category consists of a variety of cable seismic acquisition systems, which are mainly used for the shallow active surface wave survey. Examples are the 428XL series seismic data acquisition system that is produced by Sercel in France and the SUMMIT seismic data acquisition system that is produced by DMT in Germany [16,17]. Such seismometers have higher natural frequencies, satisfactory data accuracy, and lower costs; however, they use cables as the data transmission medium, which occupy more than 70% of the weight of the exploration system, reduce the convenience of instrument laying and increase the difficulty of large-scale array measurements substantially. The third category consists of all types of broadband seismographs, which have a low natural frequency, high manufacturing cost and heavy weight (all weight more than 10 kg) [18]. They require independent global positioning system (GPS) modules, recorders, and supporting power supplies, which cause inconvenience in transportation and portability [19,20,21]. Therefore, it is difficult to ensure the reliability of the field layout and perform rapid field applications of MSM with dense array measurements. Since MSM utilizes natural noise, the signal is weak, and it is particularly susceptible to strong surrounding vibration noise, which imposes higher requirements on the weak signal detection capability of the instrument in a background with strong interference [22]. It may be cost-prohibitive to replace exhausted batteries or even impossible in hostile environments [23]. In addition, MSM requires long-term observations for carrying out deep and high-accuracy exploration of the stratum [24]. Since the accuracy of data are important to the whole system’s performance, detecting nodes with faulty readings is an essential issue in data acquisition management [25,26]. This equipment cannot meet the requirements of long-term data acquisition and the quality of the signal cannot be guaranteed due to the blind acquisition mode [27]. A wireless data quality monitoring system on the acquisition system for observing the data quality during the acquisition process is urgently needed.

The main objective of this paper is to overcome the challenges that are discussed above by developing an integrated energy-efficient wireless sensor node that realizes high data accuracy for MSM. This paper considers high-precision data acquisition system design, energy-efficient mode design, and a wireless online real-time data quality monitoring system for each sensor node in the microtremor exploration. Each node adopted an integrated design, combined with three 2 Hz moving coil geophones and a high-precision acquisition system for collecting ambient noise in three directions using three channels. We utilized a high-precision analog-to-digital conversion chip and noise matching technology to realize the low-noise design that is required by MSM. A 4G wireless data quality monitoring system was built on the node. The control center used the 4G wireless monitoring system to perform real-time status configuration and data sampling display, which overcomes the closed technical defects. In addition, DFS and DPM technologies were used to reduce the system power consumption and prolong the field operation time. The proposed system is tested in both the laboratory and the field. The system that is presented in this paper can be considered a substantial upgrade and improvement on the present equipment for MSM.

The remainder of this paper is organized as follows: The recent applications of the microtremor survey method and instruments are briefly discussed and reviewed in Section 1. Development of the integrated sensor node for MSM with a low-noise design, a wireless data quality monitoring system design, and an energy-efficient design is discussed in Section 2. The results of system tests, including a noise level test and a power consumption test, and instrument performance index comparisons are presented in Section 3. In addition to the results that are presented in the end of Section 3, field measurements are provided to demonstrate the performance of the 4G wireless data quality monitoring system. The conclusions of this work are discussed in Section 4. 

## 2. Development of the Integrated Wireless Sensor Node

**Figure 2:**
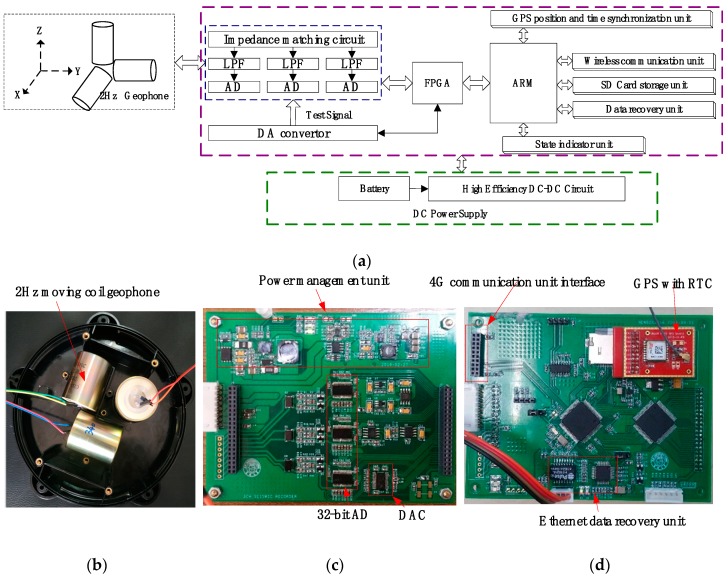
Integrated wireless sensor node for MSM. (**a**) The design scheme and pictures of the system prototype. (**b**) The top view of the three 2 Hz moving coil geophones in the orthogonal arrangement. (**c**) The top view of the acquisition and power management circuit with the PCB. (**d**) The top view of the FPGA and ARM Control system circuit with the PCB.

Based on the principle of MSM, the system structure design of the integrated wireless sensor node is illustrated in Figure 2. It consists of three 2 Hz low-frequency moving coil geophones, a data acquisition unit, and a power management unit. The node adopts a data acquisition unit with a 32-bit high-resolution ADC, namely ADS1282 (produced by Texas Instruments, Dallas, TX, USA), as the acquisition core and FPGA with the Cyclone IV(produced by Altera, San Jose, California, USA) version as the acquisition and test controller. A single-chip test signal generator, namely, DAC1282 (produced by Texas Instruments, Dallas, TX, USA), is also integrated on the acquisition unit. It can realize three-directional data acquisition, conversion, transmission, and channel testing for weak signals in MSM. A high-performance microcontroller, namely, STM32 F407, which is based on the ARM Cortex-M4 kernel (produced by the STMicroelectronics Company, Geneva, Switzerland), is used as the CPU. The CPU combines an SD card storage unit, a GPS positioning and time synchronization unit, a low-power 4G wireless communication unit, and a wired ethernet unit for data storage, data quality monitoring, and data recovery.

The instrument has a built-in GPS module in each node for receiving GPS satellite signals during data acquisition. The GPS satellites are loaded with high-precision helium atomic clocks for providing accurate coordinated universal time (UTC) time information on a global scale. After receiving the GPS information, the instrument converts the GPS data to a common time and position information, then saves the position information to the SD card. For energy-efficient design of the instrument, we combined GPS and a real-time clock (RTC) for acquiring time information. The power consumption of the GPS is far higher than that of the RTC. Therefore, RTC was used to timestamp the data file. To reduce the time error, GPS is used to synchronize the RTC time every five minutes. An SD card is used for data storage, which has the characteristics of small size, large storage capacity, and fast data storage speed. The SD card stores the microtremor data that has been signed by the RTC every whole second and the time information of the first data point in the data file that corresponds to the file name in the time domain.

### 2.1. Low-Noise Design

According to Gutenberg’s theory of wave magnitude, taking a magnitude of −2 as an example (the earthquake signal amplitude is approximately 4.7 μV), to record clear microtremor signal, the following formula should be satisfied:

(1) $$\frac{C(1-\frac{S}{P})}{T}>3$$

where C denotes the sampling rate, T denotes the highest frequency of the collected signal, S denotes the front-end noise of the instrument and P denotes the voltage amplitude, which is converted from the vibration signal at the measurement point.

According to Equation (1), there are two ways to improve the data accuracy of the instrument: Reducing the front-end input noise level of the instrument and increasing the sampling rate [28]. In the process of instrument development, the two approaches are mutually constrained. With the increase of the sampling rate, the front-end input noise of the instrument will also increase. Therefore, we must choose an optimal joint point, which can improve the noise level of the instrument. We obtained the results that S<1.88 μV when C=100 Hz and T=20 Hz as an example according to Equation (1). That is, the input noise of the front-end of the instrument is less than 1.88 μV, which ensures that more than three effective points can be collected in each cycle and the data waveform can be identified clearly.

External interference, power supply interference and channel noise are major instrument analog channel noise sources. External interference is overcome mainly by improving the PCB design, e.g., via the addition of shielded metal shells, and the planning of the power supply ground circuit. In addition, all integrated circuit pins must be properly connected to the power supplies. These connections, including pads, tracks, and vias, should have an impedance that is as low as possible, which is typically realized via the use of a thick track width and, preferably, the use of dedicated power supply planes in multilayer PCBs [29]. To reduce the system power interference, the analog circuit is completely isolated from the digital circuit power supply portion, thereby reducing the interference of the digital circuit to the analog acquisition circuit, and the digital device is powered by a separate circuit. A PCB layout must separate the circuits to reduce cross-coupling on the PCB according to their EMI contributions. Each block (noisy, low-level sensitive, or digital) should be grounded individually and all grounds should return a single point. The analog channel’s own noise comes mainly from the amplifier circuit. When the external resistor is not considered, the total equivalent input noise of the op amp circuit comes from three sources: The op amp input voltage noise, the op amp input current noise, and the thermal noise of the external source resistance. The equivalent noise of the three noise sources can be expressed as Equation (2) and the resistance thermal noise can be expressed as Equation (3).

(2) $$e=\sqrt{e_{n}^{2}{+(i}_{n}R_{s}{)}^{2}{+V}_{\mathrm{RS}}^{2}}$$

(3) $$V_{\mathrm{RS}}=\sqrt{{4\mathrm{KTR}}_{S}}$$

where $e_{n}\;$ denotes the input voltage noise of the amplifier, $i_{n}\;$ denotes the input current noise of the amplifier, $V_{\mathrm{RS}}$ is thermal noise of the external source resistance, $R_{S}$ is the external source resistance, K is the Boltzmann constant, and T is the absolute temperature, which is typically 290 K. According to the optimal noise matching principle of the amplifier, the amplifier has the smallest noise figure only when the external source impedance, namely, $R_{S}$, and the optimal source resistance, namely, $R_{\mathrm{SO}}$, are equal. If they are equal, the optimal noise performance is realized, and the minimum noise figure is obtained.

**Figure 3:**
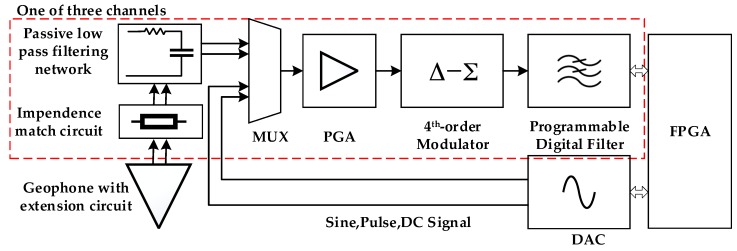
Block diagram of the acquisition board. Each node contains three duplicate channels and only one of them is illustrated in the dashed box.

The sensor node adopts three orthogonal high-sensitivity moving-coil geophones and the thermal resistance noise in the moving coil is approximately 0.8 nV when the sensitivity reaches 2 V/cm·s^−1^. The acquisition channel circuit is responsible for completing the signal matching, buffering, filtering, and amplification and, finally, converting the analog signal to the digital signal. The developed structure of the data acquisition system is illustrated in Figure 3. Each of the three channels is composed mainly of an input protection circuit, an impendence matching circuit, a passive low-pass filter circuit, a programmable voltage gain amplifier, a fourth-order Δ-∑ modulator and a programmable digital filter circuit. The microtremor raw signal is filtered by a passive low-pass filtering network, which includes a differential filter and two independent filters. The differential filter removes high-frequency normal mode components from the input signal. Independent filters remove high-frequency components that are common to both input signals (common-mode filter). A high-accuracy 32-bit Δ-∑ ADC ensures outstanding overrange detection noise and the linearity of the data acquisition performance. △-∑ is a type of ADC that combines oversampling technology with noise-shaping technology [30]. The oversampling technology distributes the quantization noise to a higher bandwidth, which reduces the quantization noise in the original bandwidth and reduces the distortion of the signal. A test system that consists of a high-performance digital-to-analog converter, namely, DAC1282, is used to test the acquisition channel. The mux switch switches the acquisition channel to the signal test channel when the sensor node is powered on. In the process of channel test, MCU controls the DAC1282 to generate a sine wave or pulse test signal, then detects the related index parameters of the acquisition system.

### 2.2. Wireless Data Quality Monitoring System Design

Typical cable-less seismographs do not have a communication cable for transmitting seismic data. A data recorder stores the data in the built-in memory and a data recovery system is used to collect the data from all the recorders for the host when the acquisition task has been completed [27]. This method cannot be used to perform real-time quality monitoring of the working status of each recorder and the construction quality and efficiency are difficult to guarantee. Many seismographs only transmit commands via a simple wireless monitoring system; therefore, it is difficult to ensure the quality and reliability of the data. The data packet loss rate is higher under electromagnetic interference or obstacle obstructions. Data resending leads to a lower data transfer rate and affects the efficiency of the collection operation. Therefore, the wireless data quality monitoring system is one of the key technologies that determine the performance of microtremor exploration instrument system. Microtremor data acquisition will generate a large amount of data for long-time observation and data processing is not performed in real time. Wireless real-time data recovery will consume a large amount of power. When data are transmitted wirelessly, there are strict requirements on bandwidth of the wireless system. Therefore, massive data are stored directly on the built-in large-capacity SD card. To ensure the construction quality and to satisfy the data quality requirements of MSM, this paper constructs a 4G wireless monitoring system on the node and uses the data sampling test method to monitor the data in real time. In actual microtremor exploration, the total amount of data that is transmitted on a single line can be expressed as Equation (4).

(4) S=N×C×fs×t×B

(5) S=N×C×fs×t×B×q

where N denotes number of nodes on the line, C denotes the number of acquisition channels per node, fs denotes the sampling rate, t denotes the data recording time and B denotes the size, in bytes, that is occupied by each sample point. This node uses three acquisition channels and each of them has a 32-bit AD; thus, the total amount of data for each instrument is 6 Kbytes at a 500 Hz sampling rate. When multiple nodes perform wireless data quality detection at the same time, the transmission load becomes large and data congestion occurs. A large amount of packet loss can be generated, which increases the propagation delay and the energy consumption of the node, which directly affects the performance of the entire exploration system.

Combined with the characteristics of the signal, this paper uses the digital decimation filtering method to extract and filter the signal that is collected at the normal sampling rate, thereby reducing the amount of transmitted data and, thus, substantially reducing the occurrence of data congestion. If the data extraction factor is q, the total size of the data that are transmitted on a survey line will be expressed by Equation (5). The value of q is between 0.1−0.2 and the amount of collected data becomes 600 bytes or 1.2 Kbytes at a 500 Hz sampling rate after decimation and filtering.

**Figure 4:**
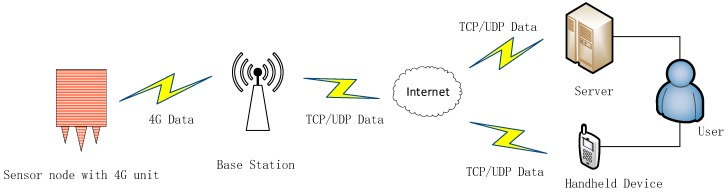
Diagram of the 4G module application in each node.

Considering the transmission distance, power consumption, and data transmission rate, the node adopts a low-power remote transmission 4G module, namely, USR-LTE-7S4 (produced by USRIOT company, Jinan, China), which is suitable for the 4G network standard and transparent transmission of data between node devices and network servers via simple instructions. The 4G network transmission rate exceeds 50 Mbps, which meets the requirements of a wireless quality monitoring system with more than 1000 nodes. When there is no 4G network environment, the system can seamlessly switch to the 3G/2G network. A 4G module application diagram is shown in Figure 4.

**Figure 5:**
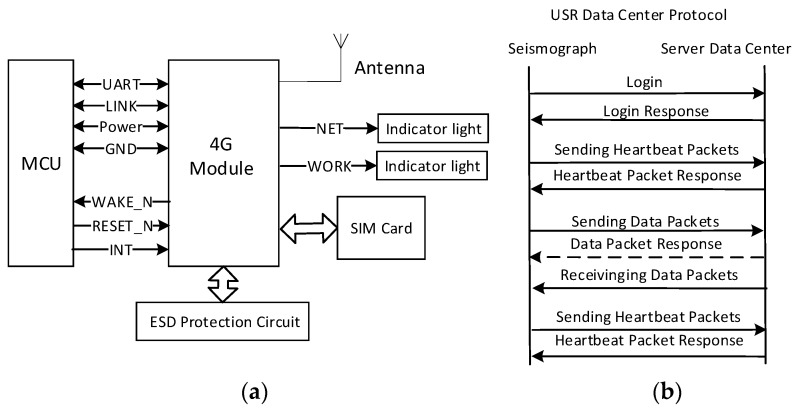
Diagram of the 4G module. (**a**) A system block diagram of the 4G module interface. (**b**) The UDCP interaction flow chart.

A system block diagram of the 4G module interface is shown in Figure 5a. When the node is in a low-power sleep state, the 4G module wakes up the node via the WAKE_N pin. The RESET_N pin is used to reset the 4G module. When the data transmission fails, the host resets and restarts the 4G module via the RESET_N pin. The 4G module provides an interrupt INT pin via which the seismometer can quickly respond to notification requests for the control center. In the standard mode, the 4G module provides a 3.25 MHz clock signal to the SIM card. In the low-power mode, the SIM card is provided with a 1.08 MHz clock signal and in the logout mode, the clock signal is turned off and the automatic power saving mode is entered. The blinking frequencies of the indicator unit that is connected to the NET pin represent the current network status. To prevent static electricity damage to the SIM card and the chip, a TVG tube is added to the 4G module for electrostatic protection as an anti-static measure. This paper defines a user data center protocol (UDCP) that is based on private registration packets and heartbeat packets for stable and reliable data transmission. The UDCP interaction flow chart is shown in Figure 5b. UDCP defines a byte-stream data format that provides login, heartbeat, data transfer, and exit mechanisms that are based on UDP and TCP. As shown in Figure 5b, the data interaction is divided into three phases:

1. Login: The node must log in to the center prior to sending the data. If the login is successful, the heartbeat will be sent at a specified interval. If the heartbeat response is not received for a specified consecutive number of times, the connection is deemed incorrect. The login process will be reinitiated and restarted if necessary.
2. Data transmission: Data transmission can be divided into the request-response mode and the active reporting mode, namely, the node can actively report data and the center can also send data actively.
3. Logout: An attempt is made to send an active offline packet prior to disconnecting the network. However, since the network is often unreliable at this time, the packet may be lost. The service center relies not on the data packet for judging the terminal status, but on the heartbeat timeout.

**Figure 6:**
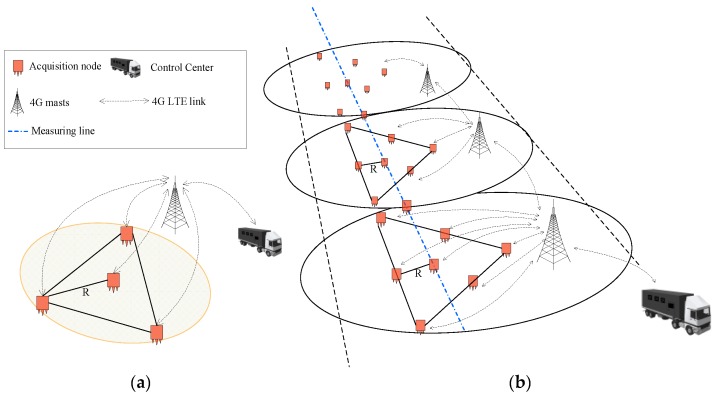
Observation array for MSM (**a**) single-point detection and (**b**) 2D measurement microtremor profiling.

**Figure 7:**
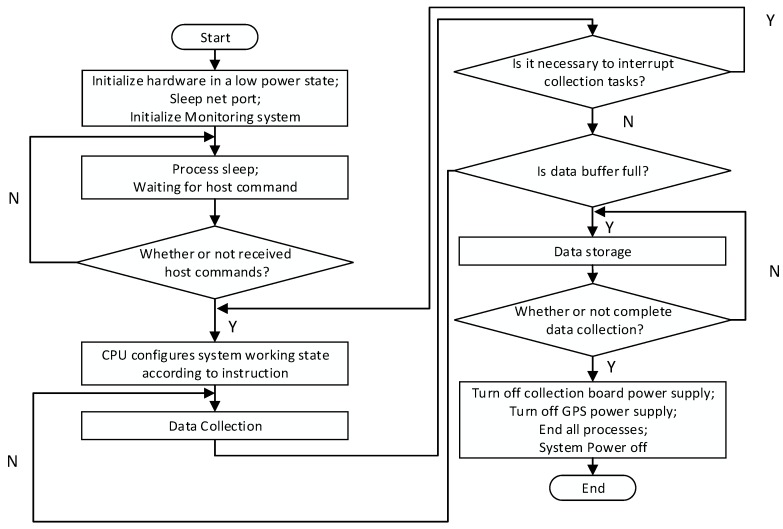
Flow chart of the complete microtremor data acquisition process.

Typically, MSM has an observation depth of approximately several kilometers; hence, the radius of the observation array is between several meters and several kilometers. When using the SPAC method, it is necessary to arrange the stations along the circumference with at least three stations at equal intervals on the circumference and one station at the center of the circle to form a circular observation array (see Figure 6a). The radius of the circular array is called the observation radius, which determines the depth of detection. Typically, the detection depth of the microtremor array is 3−5 times the observation radius. It is often necessary to use multiple circular arrays for combined observations to form a two-dimensional observation system, as shown in Figure 6b. The control center sends the command to the acquisition node through the 4G wireless system. After receiving the command, the acquisition system collects real-time data in addition to the normal storage to the SD card and subsequently down-samples the data. Then, it sends the data to the control center via the 4G wireless network. Field operators monitor the quality of the data accurately and timely according to the waveform display and determine whether the data acquisition state is abnormal. A flow chart of the complete microtremor data acquisition process is shown in Figure 7.

Prior to the data acquisition process, the control center performs unified state configuration, e.g., of the sampling rate, acquisition channel gain, and acquisition channel filter parameters, on all online nodes via the 4G wireless system. During the data acquisition process, the collection unit is awakened for data collection by the wireless monitoring system. The sensor node performs real-time sampling detection on the currently collected data and combines the wireless quality monitoring system with the local data storage to improve the data transmission reliability. If a fault is encountered during the data acquisition process, e.g., if the external environment has a large vibration or faulty nodes are identified, thereby resulting in failure to obtain an effective signal, the server sends a sleep command to all nodes to turn off the data acquisition unit; then, the nodes enter a low-power state. After the fault has been removed, the data acquisition unit is awakened to continue the acquisition process until the data acquisition is complete. After the data collection process has been completed, the server sends a shutdown command to all online nodes through the 4G wireless monitoring system. The microtremor data are completely stored in the SD card for subsequent data recovery. Command interaction is carried out in the collection unit and the real-time data waveform is displayed on the control center side. The command interaction includes the node sampling rate, the query and setting of the gain, and the control of the running state of the collection station (including starting acquisition, stopping acquisition, sleeping, and waking). This 4G wireless data quality monitoring solution realizes on-site real-time monitoring of the quality of acquired data and ensures construction quality and efficiency.

### 2.3. Energy-Efficient Design

Most integrated circuits (IC) in seismic instruments belong to CMOS circuits because of their high integration and low power consumption [31]. The power consumption of CMOS circuits can be divided into static power (Ps) and dynamic power (Pd). Static power is also called leakage power. CMOS circuits have a quiescent current when they are not in operation mode and the quiescent current can cause static power consumption. The transistor channel reverse bias diode leakage current and the subthreshold leakage current of the shutdown device are two types of quiescent current. The reverse bias diode leakage current is generated if one transistor in the CMOS circuit is turned off and the other transistor is in the on state with charging by the potential difference [32]. The leakage current, which is denoted as $I_{L}$, can be expressed as Equation (6).

(6) $$I_{L}=A_{D}\cdot J_{S}$$

where $A_{D}$ denotes leakage diffusion area and $J_{S}$ denotes the leakage current density, which is related to the temperature as follows: The higher the temperature, the higher the leakage current density. The subthreshold leakage current is generated when the device is switched off if the applied voltage, namely, $V_{\mathrm{GS}}$, is less than the threshold voltage, namely, $V_{t}$, of the transistor, which is referred to as weak inversion mode. Subthreshold current flows due to the diffusion current of the minority carriers in the channel of the metal oxide semiconductor field effect transistor (MOSFET). This current can be expressed as Equation (7).

(7) $$I_{\mathrm{sub}}=k_{3}e^{k_{4}v_{\mathrm{dd}}}e^{k_{5}v_{\mathrm{bs}}}$$

(8) $$\mathrm{Ps}=V_{\mathrm{dd}}I_{\mathrm{sub}}+V_{\mathrm{bs}}I_{L}$$

where $k_{3}\;,k_{4},$ and $k_{5}$ are constants, $V_{bs}$ is the substrate bias and $V_{\mathrm{dd}}\;$ is the supply voltage. In summary, the total static power consumption, namely, $P_{S}$, can be expressed as Equation (8). The dynamic power consumption, namely, $P_{D}$, of the system accounts for approximately 90% of the total power consumption, namely, $P_{t}$, and it can be expressed as Equation (9). Therefore, $P_{t}$ can be expressed as Equation (10).

(9) $$P_{D}=\alpha \times C_{L}\times V_{\mathrm{dd}}^{2}\times f$$

(10) $$\mathrm{Pt}=V_{\mathrm{dd}}I_{\mathrm{sub}}+V_{\mathrm{bs}}I_{L}+\alpha \times C_{L}\times V_{\mathrm{dd}}^{2}\times f$$

**Figure 8:**
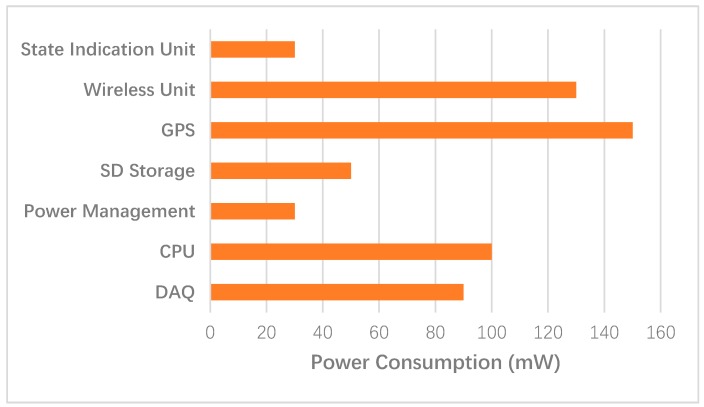
Power consumption proportion diagram of the integrated wireless sensor node.

According to Equation (8), the substrate voltage and the supply voltage are the main factors that affect the static power consumption. In practical applications, the static power dissipation increases with the heating of the device and as the device size decreases. Therefore, in the optimization of the static power, the system structure is mainly considered in selecting a series of low-power components, simplifying the components, and building a relatively simple circuit structure. The power supply voltage and the operating frequency are the main factors that affect the dynamic power consumption. To reduce the dynamic power consumption of the circuit, we initially considered the supply voltage and the operating frequency. We adjusted the working voltage for a module or device and even cut off the voltage of an unused module temporarily in the circuit dynamically. In addition, according to the operating state of the system, an unsuitable peripheral clock can be turned off or its frequency can be reduced, thereby reducing the dynamic power consumption of the entire circuit. An energy-efficient wireless sensor node is composed of a power supply, an acquisition system, and a control system, according to Figure 2a. The acquisition system includes a data acquisition (DAQ) unit and a test controller system. The control system includes a CPU, an SD storage unit, a GPS, a data recovery unit, a wireless data quality monitoring unit, and a status indication unit. Depending on the requirements of the microtremor exploration system, in the absence of real-time data transmission, a large amount of microtremor data must be stored on the SD card; thus, the power consumption of the data recovery unit is temporarily ignored. The power consumption proportion diagram of each part in the node is shown in Figure 8.

As shown in Figure 8, the GPS unit, CPU, and wireless communication unit consume the largest amounts of power and together account for approximately 62% of the total power. Designing a low-power mode for high-power-consumption units can reduce the static power consumption effectively. Since the 4G wireless communication unit is in a low-power state during the seismic acquisition process, it enters the normal operating state only when the initial state configuration and the acquisition task are faulty; hence, the power consumption of 4G module is neglected temporarily. 

STM32F-series chips reduce the power consumption of the system by ensuring high-performance data acquisition; however, the power consumption of STM32F1 processors reaches 500 μA/MHZ, which is far higher than those of the STM32F2 and STM32F4. STM32F4 adopts the Cortex-M4 kernel, enhanced DSP processing instructions, additional storage space, extreme speed, and high-performance peripherals to improve its performance compared with the STM32F2 by 25%–35% and its power consumption from the original 188 μA/MHz to 140 μA/MHz; its power consumption reduced by approximately 25% [33]. In this paper, STM32F407 is selected as the CPU and the main frequency in the processor can be adjusted to reduce the power consumption according to the operating conditions. The low-power mode of STM32F407 is divided into the sleep mode, stop mode, and standby mode. Various state configurations of CPU are used to optimize the power consumption and the optimal performance according to the operating mode.

**Figure 9:**
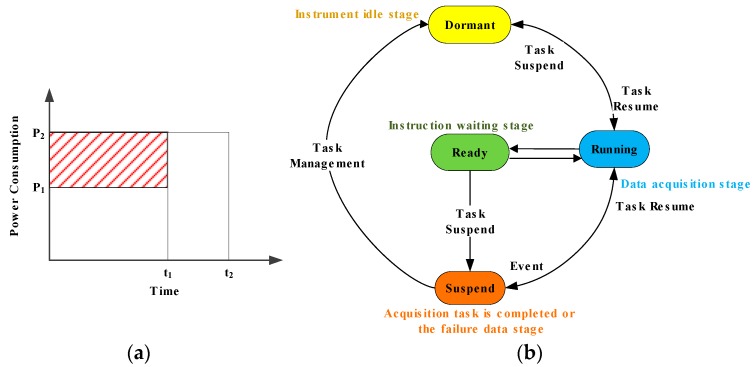
Low-power-consumption design. (**a**) A schematic diagram of the dynamic frequency selection technology (DFS) technology in MSM and (**b**) a state transition diagram of task scheduling in the dynamic power management technology (DPM) system.

Low-power-consumption optimization can be divided into dynamic frequency selection technology (DFS) and dynamic power management technology (DPM). DFS reduces the power consumption of the system by adjusting the operating frequency of the CPU dynamically according to the detected system information and constraints. During the acquisition process, the peak processing speed of the CPU is much higher than the average processing speed; hence, it is possible to adjust the power consumption and performance of the system. The realization of dynamic frequency adjustment technology includes load forecasting and adjustment judgment. In the process of data acquisition, load forecasting is mainly based on tasks and it requires a task queue that is provided by the operating system to obtain the system load in the future. The system load and operating frequency can be adjusted to the optimal state by further adjusting the operating system kernel according to the load requirements. The adjustment judgment is mainly aimed at nonreal-time systems during data collection. In nonreal-time systems, it is impossible to predict the completion time of a task; therefore, the design principle enables the system to run at a low speed according to the operating state of the system. The power consumption of the CPU is directly proportional to the operating frequency. DFS technology adjusts the operating frequency of the CPU and other peripherals as much as possible by detecting the operating state of the system to reduce the power consumption. Figure 9a is a schematic diagram of the process of reducing the power consumption of the data acquisition system using DFS technology in MSM.

In Figure 9, P_1_ denotes the CPU power consumption when it is in a low-frequency state that is adjusted by DFS, P_2_ denotes the CPU power consumption at full-speed operation, t_1_ denotes the data acquisition time and t_2_ is the time that is spent on a complete acquisition task, including data acquisition and data recovery. Using DFS technology to adjust the operating frequency of the CPU and other peripherals reduces the power consumption of $t_{1}\times (P_{2}-P_{1})$. The dynamic power management (DPM) technique saves power by configuring the system state dynamically to accomplish the system acquisition tasks. It aims at fully utilizing system idleness to switch a part of the system, e.g., the CPU, GPS, wireless communication unit or other peripheral, to a low-power or off state. A typical task state transition model is shown in Figure 9b. The power consumption of the running state exceeds that of ready state and the power consumption of the ready state exceeds that of the hanging state. According to the flow chart of the acquisition process in Figure 7, the operating mode of the sensor node is divided into the instruction waiting stage, the data acquisition stage, the instrument idle stage, and the failure data stage. Each peripheral unit has operating states that differ among the operating stages. In the instruction of the waiting stage, only the wireless module is opened, and the controller and other units are in a dormant state. If the sensor node receives the state configuration instructions, the operating mode is open, and data are collected. When the acquisition has been completed or failure data are identified and intermittent time interval acquisition occurs, the control center switches the sensor node into dormant mode via the wireless configuration thus that most of the peripherals of the system are in low-power consumption states. When the system recovers, data collection is continued until the end of the entire acquisition process. However, in practice, the peak power consumption of each module is much larger than the average power consumption in the various operating states. If the entire high-speed operation mode is adopted, the CPU and part of the hardware will be idle most of the time and a substantial amount of energy will be wasted in idling. When the system does not accept the processing task command, it can switch from the running state to the suspended state or dormant state to reduce its power consumption according to the requirements.

## 3. System Testing and Field Measurement for Validation

### 3.1. Noise-Level Test

**Figure 10:**
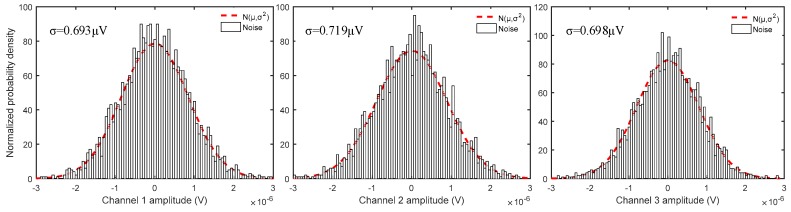
Distributions of the noise at the front-end of the instrument.

The low-noise test, which is discussed in Section 2.1, is implemented in the laboratory using a sensor node and 64-bit MATLAB. This program is capable of carrying out the high-data-accuracy tasks that are required by MSM using the high-precision AD chip and noise-matching technology. The lower the equivalent input noise, the higher the data accuracy. We evaluated the inherent noise properties of the receiver system by shorting the input to each preamplifier and storing the data in the SD card. The instrument uses a short-circuit method at the front-end to perform the equivalent noise test. The voltage at the output is divided by the amplification factor of the entire instrument system. Then, the result is the equivalent input noise. Figure 10 shows the test results of the input noise at the front-end of the instrument.

Figure 10 presents the equivalent noise output results of the three channels with 0 dB gains and a 500 Hz sampling rate. According to the obtained results from the processed data, when the amplification of the programmable amplifier was 0 dB, the equivalent input noise of the three channels was 0.693 µV, 0.719 µV, and 0.698 µV. Therefore, the acquisition accuracy of the sensor node satisfies the expected requirements.

### 3.2. Power Consumption Test

As discussed in Section 2.3, the effect of the energy-efficient design is investigated in the laboratory using a sensor node and a multimeter. This program can carry out the long-term observation tasks that are required by MSM using the DFS and DPM technologies. The operating state of each module of the sensor node in each operating mode and operating period is listed in Table 1. Each module is set to low-power mode or sleep mode in the non-operating state to reduce the average power consumption of the system and each module is awakened when necessary.

As the CPU dynamically adjusts the operating frequency of the chip and the modules of each peripheral according to the system tasks, STM32F407 enables the system to reduce its power consumption while ensuring that the acquisition task is completed. While waiting for the wireless state configuration phase, the frequency of the central processor is adjusted to 20 MHz in the system acquisition state and the wireless module is turned on while waiting for the control center to perform the instrument state configuration. In the data acquisition periods, the wireless module is placed in sleep mode to reduce the power consumption. When a fault is encountered, the system enters a standby state and the wireless module, and the acquisition unit are placed in a low-power mode. In the data recovery stage, the wired ethernet module is turned on for data recovery, while other modules are hibernated to realize low power consumption. The sensor node is powered by lithium batteries and it can operate continuously for more than 30 days in continuous acquisition mode. Therefore, the power consumption of the sensor node satisfies the expected requirements.

### 3.3. Performance Comparison

Contrast key technical indicators would be more convincing to validate the reliability of the sensor node, the wireless microtremor acquisition system was compared with a popular seismic acquisition instrument on the market. The selected acquisition instrument was ZL and 3C, which is produced by the Fairfield Nodal Company. Twelve aspects, including the equivalent input noise, data monitoring, operating life, and dimensions, were compared. The superior performance of the high-precision wireless sensor node is reflected in some of these aspects. Table 2 lists the performance comparison results.

### 3.4. Field Measurement

**Figure 11:**
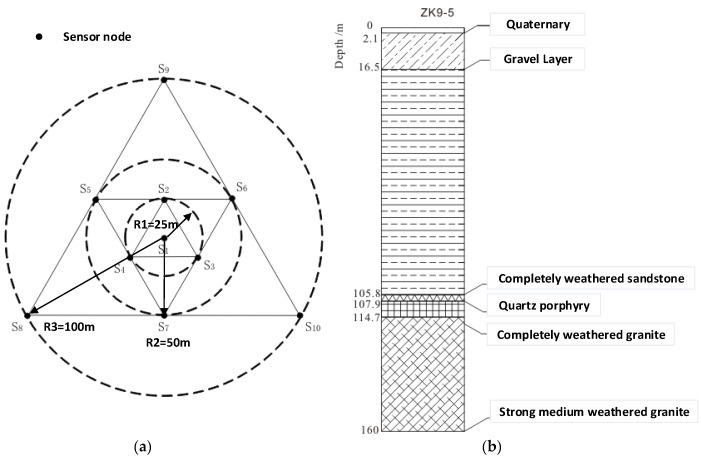
(**a**) The layout of the microtremor observation site. The small black circles on the triangles and their centroids represent sensor nodes of the survey point. (**b**) Stratigraphic and lithological logs of the ZK9-5 borehole that is located at survey point S1.

To evaluate the reliability and performance of the 4G wireless data quality monitoring by the sensor node for microtremor investigations, a field microtremor survey was conducted at Feng Zhen City, which is in the Inner Mongolia Autonomous Region of China. At the survey location, a nested triangular array with ten sensor nodes was used, as shown in Figure 11a. We placed a sensor node at the center of the triangle and the other nodes in a circle of radius R. The smallest array has a radius of 25 m and the largest array has a radius of 100 m. A sampling rate of 500 Hz was adopted, and the exploration tasks took four hours. According to the previous drilling results, which are shown in Figure 11b, the interior layer structure of the field is simple. A quaternary new alluvium sandy soil and gravel layer, which extends approximately 16.5 m with a covering loess layer of 2.1 m, is the main component of the upper part of the accumulation layer. The middle layer of the exploration area is composed of completely weathered sandstone and the original rock structure has been destroyed. The completely weathered sandstone has weathered away into sandy soil with a small amount of debris and poor mechanical strength. The bottom layer is composed of completely weathered granite, which is massive and columnar. The rock fragmentation is mostly fragmental with typical mechanical strength.

**Figure 12:**
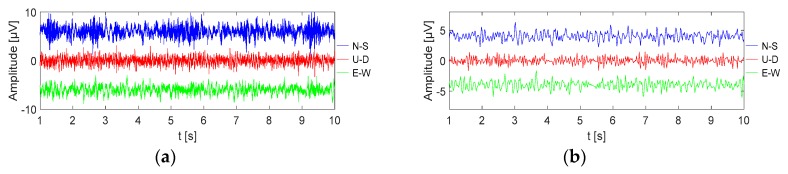

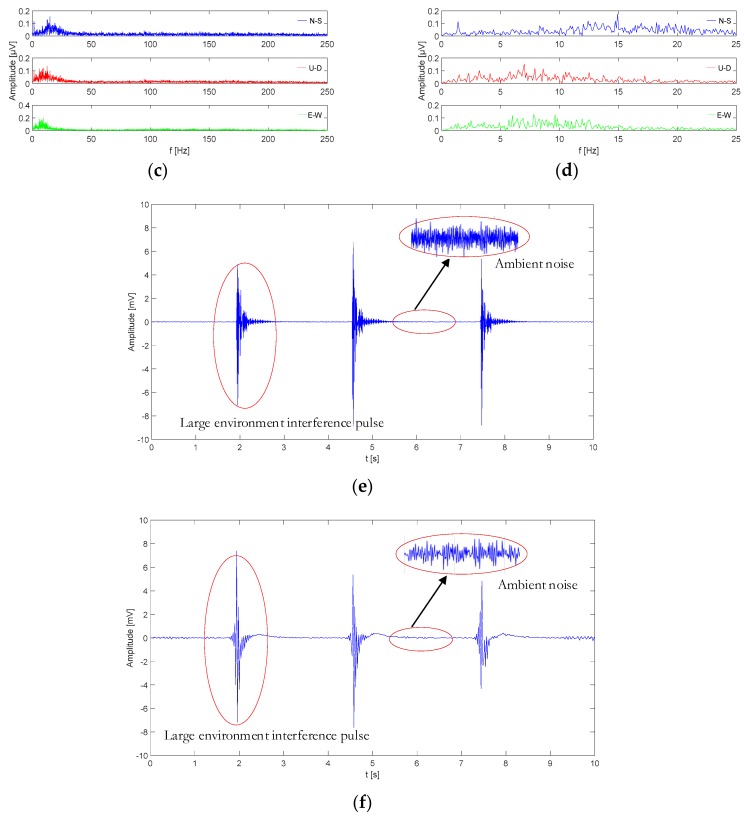
Example data records at survey point S1. (**a**) Three-component microtremor data from survey point S1. (**b**) The three-component microtremor data that are recovered by the wireless monitoring system through the control center when the data extraction factor is 0.1. (**c**) Typical recordings in the frequency domain for three-component microtremor data. (**d**) Typical recordings in the frequency domain for three-component microtremor data that are recovered by the wireless monitoring system through the control center. (**e**) Large environmental interference into the process of data collection and (**f**) large environmental interference into the process of data collection by the wireless monitoring system through the control center.

According to Figure 12c,d, the normal data only contain low-frequency signals. Figure 12b,f show that the wireless monitoring system can monitor the normal operation of the data acquisition work. To evaluate the performance of the wireless monitoring system, we use a hammer signal to simulate large environmental interference. The wireless monitoring system can identify the fault and halt the acquisition work when large environmental interference is encountered. The data processing method of MSM is based on the stationary stochastic theory. A digital signal processing technique was used to extract the fundamental surface wave from the ambient noise and the phase velocity dispersion curve is measured to obtain the S-wave velocity structure below the survey area. The data recording for a period can be regarded as a stationary stochastic process. The data records that are obtained by each sensor node are divided into several data segments to remove the large interference and the spatial autocorrelation function between the nodes is calculated. 

**Figure 13:**
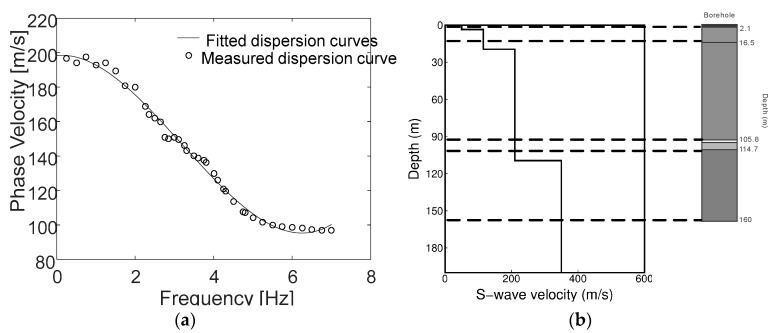
(**a**) The shear-wave phase velocity dispersion curves that were obtained via MSM and (**b**) the apparent S-wave velocity structure of the observation site.

By fitting the autocorrelation coefficients of instruments at various distances at the same frequency, the phase velocity dispersion curves of the formation below the array can be obtained, as shown in Figure 13a. From the obtained dispersion curve of the subsurface medium, the apparent S-wave velocity, which is denoted as $V_{x}$, can be calculated and the $V_{r}-f$ curve can be converted into a $V_{x}-H$ curve via Equation (11).

(11) $$V_{x,i}={\left(\frac{t_{i}\times V_{r,i}^{4}-t_{i-1}\times V_{r,i-1}^{4}}{t_{i}-t_{i-1}}\right)}^{\frac{1}{4}}$$

where $V_{r}$ is the Rayleigh wave velocity and $t_{i}$ is the cycle. Finally, the calculated $V_{x}-H$ curve is obtained via fitting and interpolation to obtain a structure, as shown in Figure 13a.

The loose quaternary alluvial sand and gravel layers correspond to the low-velocity zone at the depth of 20 m in the apparent S-wave velocity structure curve. At approximately 100 m depth, a moderately weathered sandstone layer is demonstrated by the substantial velocity difference. The apparent S-wave velocity structure that was obtained from the field measurements was consistent with the corresponding stratum data that were obtained from the borehole. The sensor node that was developed in this paper captured the microtremor signal and, thus, obtained the information of the deeper layers effectively. This node has the characteristics of low energy consumption and wireless monitoring, which are convenient for large-scale high-density layout exploration. 

## 4. Conclusions

In this study, a high-precision integrated energy-efficient wireless sensor node has been designed for MSM. The system design utilized the three channels to collect microtremor signals in three directions. At the same time, a low-noise design that satisfies the requirements of MSM has been realized based on noise-matching technology and the high-precision 32-bit AD chip. Furthermore, the acquisition system uses the data quality monitoring system and the real-time state configuration, control, and data sampling display of the sensor node through the 4G wireless monitoring technologies to overcome the closed technical flaws. Finally, a power analysis was conducted on the instrument. DFS and DPM were used to reduce the power consumption. DFS changes the operating mode or operating frequency of the peripherals according to the operating state of the system and adjusts the operating frequency of the master chip dynamically. Under the premise of ensuring various system performance parameters, the DPM strategy was formulated for improving the energy utilization of high-energy-consuming modules effectively.

The results of the in situ testing and field measurement of the integrated sensor node demonstrated that the sensor node met the expected performance requirements on the equivalent noise, data monitoring function, and power consumption. Thus, the integrated energy-efficient wireless sensor node ensures the reliability of the field layout and enables rapid field applications of MSM with dense array measurements.

## Figures and Tables

**Table 1:** Power consumption measurements during various operating periods.

Stage	DAQ	CPU	GPS	SD	Wireless	Ethernet	Power
Instruction idle stage	#	√*	#	#	√	#	90 mW
Standby stage	√*	√*	√*	#	√	#	150 mW
Data acquisition stage	√	√	√	√	√*	#	220 mW
Failure stage	#	√*	#	#	√	#	90 mW
Data discovery stage	#	√	#	#	#	√	320 mW

√ Operating mode √* Low-power mode # Sleep Mode.

**Table 2:** Instrument performance index comparison results.

Indicator	The Proposed Sensor Node	ZLand 3C
Sensor	3 Geophones, Orthogonal Configuration, 2 Hz–70% damped, 2 V/cm/s	3 Geophones, Orthogonal Configuration, 10 Hz–70% damped, 78.7 V/m/s; 5 Hz–70% damped, 76.7 V/m/s
Data Channels	3	3
ADC Resolution	32 bits	24 bits
Sample Interval	0.25, 0.5, 1, 2 and 4 milliseconds	0.5, 1, 2, and 4 milliseconds
Preamplifier Gain	0 dB to 36 dB in 6 dB steps	0 dB to 36 dB in 6 dB steps
Digital Filter	Sinc + FIR + IIR	Linear Phase or Minimum Phase
Operating Life	over 30 days, Continuous	20 days, Continuous
Equivalent Noise	0.7 μVrms @ 0 dB	0.75 μVrms @ 0 dB
Data Monitoring	4G	nothing
Timing Accuracy	± 10 μs, GPS Disciplined	± 10 μs, GPS Disciplined
Weight	1.85 kg	2.8 kg, including spike
Dimensions	12 cm diameter by 12 cm high	11.7 cm diameter by 16.3 cm high

Indicator test environment: 2 ms sampling rate, 25 °C.
